# Does Differentiated Leadership Threaten Who I Am? Introducing a Self-Verification Perspective to Explain the Curvilinear Effect of Differentiated Empowering Leadership

**DOI:** 10.3389/fpsyg.2019.01903

**Published:** 2019-08-23

**Authors:** Shaolong Li, Shudi Liao, Fang Sun, Zhiwen Guo

**Affiliations:** ^1^Economics and Management School, Wuhan University, Wuhan, China; ^2^Business School, Hubei University, Wuhan, China

**Keywords:** differentiated leadership, empowering leadership, relationship conflict, counterproductive work behaviors toward individuals, self-verification theory, team competence variance

## Abstract

Based on the self-verification theory, this research proposed a multi-level model for exploring whether, how, and when differentiated leadership had curvilinear effects on relationship conflict within a team and further on team members’ counterproductive work behaviors toward individuals (CWBI). Drawing on a sample of 297 team members nested in 78 teams, we found that differentiated empowering leadership had no direct curvilinear effects on relationship conflict. However, the results showed that the team competence variance could moderate the curvilinear relationship between differentiated empowering leadership and relationship conflict. Specifically, only in teams with high competence variance among members, differentiated empowering leadership had a U-shaped effect on relationship conflict. Moreover, differentiated empowering leadership interacted with team competence variance had a downstream effect on team members’ CWBI through relationship conflict. We ended up by discussing the theoretical and practical implications of these findings.

## Introduction

“One of the biggest factors that separate dysfunctional from high-performing teams is leadership.”

–Kirkman and Harris, 2017, p. 6

With the rise of team-oriented work structures, team leadership has attracted tremendous attention from researchers and practitioners (Day et al., 2006; Morgeson et al., 2010). A proliferation of research has studied how traditional leadership theories, such as transformational leadership (Chen et al., 2007; Eisenbeiss et al., 2008) and empowering leadership (Li N. et al., 2017), operate in a team context. However, the simple transplantation of traditional leadership theories to team situations has neglected the distinction between leader-subordinate interactions and leader-team interactions (Zaccaro et al., 2009). Thus, an area that has begun to receive increased attention is the role of differentiated leadership in team settings. For example, many researchers have explored leader-member exchange (LMX) differentiation (Liden et al., 2006; Liao H. et al., 2010; Le Blanc and González-Romá, 2012). Several other researchers have studied differentiated transformational leadership (Wu et al., 2010; Cole et al., 2011), differentiated empowering leadership (Li et al., 2015; Li S.L. et al., 2017), and differentiated laissez-faire leadership (Cole and Bedeian, 2007).

Unfortunately, this body of differentiated leadership research has not yielded a breakthrough in team leadership research, at least for now, because the existing studies did not reach consistent conclusions. Bauer and Erdogan (2015) concluded that LMX differentiation (a typical type of differentiated leadership) “is understudied and conclusive findings are hard to come by” (p. 418). Specifically, based on a fairness perspective, Hooper and Martin (2008) found that LMX differentiation decreased employees’ job satisfaction and well-being. Wu et al. (2010) concluded that differentiated transformational leadership might damage team effectiveness. In contrast, Liden et al. (2006) did not indicate support for the main effects of LMX differentiation on individual and team performance. Stewart and Johnson (2009) even found that among diverse gender groups, LMX differentiation was positively related to workgroup performance when aggregate LMX was high. The inconsistent effects of differentiated leadership might imply a potential curvilinear pattern in its nature. In other words, it may be dysfunctional for team cooperation when differentiated leadership is too high or too low. Nevertheless, as far as we know, no study has empirically explored the potential non-linear effect of differentiated leadership on team and individual outcomes.

Recognizing this interesting and important yet unaddressed issue, we investigate the curvilinear (U-shaped) effect of differentiated leadership (i.e., differentiated empowering leadership) on team process (i.e., relationship conflict) and further on team members’ behavior (i.e., counterproductive work behavior toward individuals: CWBI) in this study. Differentiated empowering leadership, defined as the degree to which team leaders give different amounts of authority and autonomy to their team members (Li et al., 2015, Li S.L. et al., 2017), has drawn increasing interest because of recent widespread advocacy for and application of empowerment in team settings (e.g., Google) (Li S.L. et al., 2017).

Existing research has found that empowering leadership can promote employee’s innovative behavior, teamwork behavior, turnover intention, proactive behavior, and organizational citizenship behavior in a team context (e.g., Chen et al., 2011; Li N. et al., 2017). However, other empirical research produced confounding results. For example, Yun et al. (2005) found that in trauma resuscitation teams, empowering leadership was less effective than directive leadership when trauma severity was high or when the team was inexperienced. Lorinkova et al. (2013) found that directive leadership initially outperformed empowering leadership. Humborstad et al. (2014) found a curvilinear relationship between empowering leadership and employee performance. Hence, several researchers have called for more exploration of the “dark side” of empowering leadership (Sharma and Kirkman, 2015; Cheong et al., 2019). We draw on the diversity perspective (Harrison and Klein, 2007) and examine whether and how differentiated empowering leadership induces detrimental outcomes in this study.

By introducing the self-verification theory (Swann, 1983) as the overarching framework, we explore relationship conflict and CWBI as dark side outcomes of empowering leadership in the team context. Increasingly, leadership researchers have claimed that self-concept is central to understanding the development of interpersonal relationships (Engle and Lord, 1997; Lord et al., 1999). In addition, several research teams have argued that the self-verification process is important in team member interactions (Swann et al., 2000, 2003, 2012; Purvanova, 2013). Relationship conflict is implicated as the extent of interpersonal relationships and has been considered as an important outcome of the self-verification process in the team context (Polzer et al., 2002). Besides, relationship conflict has been strongly supported as a detrimental factor for team operation and effectiveness (De Dreu and Weingart, 2003). In addition, several researchers have stated that team member relationship is a crucial team-level outcome of differentiated leadership (e.g., Henderson et al., 2009). Hence, we can bring new insights to the literature on differentiated leadership and empowering leadership in the team context by examining the U-shaped relationship between differentiated empowering leadership and relationship conflict.

We explore whether the curvilinear influence of differentiated leadership on team process would further induce team members’ negative behaviors. Counterproductive work behavior (CWB) is a potentially negative behavior accompanying relationship conflict (Hershcovis et al., 2007; Berry et al., 2012). However, the limited research investigating the relationship between positive leadership and CWB has produced insufficient insight into the leadership-CWB relationship (Judge et al., 2006; Holtz and Harold, 2013; Kessler et al., 2013). Compared with CWB toward organizations (CWBO), relationship conflict among team members is more likely to induce harm and damage toward other individuals (CWBI) within the team than the organization (Bruk-Lee and Spector, 2006). Accordingly, we study CWBI in particular as the downstream outcome.

Drawing from the self-verification theory, people are motivated to maintain their self-view (Swann, 1983). Receiving self-verification can smooth interpersonal interactions (Swann et al., 2000). We assume that a low or high level of differentiated empowering leadership may damage team members’ stable self-view (i.e., competence in this study), which is a crucial source of coherence, and further destroy within-team interactions (i.e., high relationship conflict) (Swann, 1983, 1987, 1990). A diverse team environment can lead to different characteristics of individuals’ self-views becoming salient (Swann et al., 2004). Accordingly, we further test and expand our framework by examining team competence variance as a reflection of the team environment and a key boundary condition. Specifically, significant variance in team competence may cause a low or high level of differentiated empowering leadership to be more likely to damage team members’ consistent self-views of competence, and thereby induce team relationship conflict. Moreover, this pernicious influence will transmit to team members’ deviant behaviors toward coworkers.

We seek to make several theoretical contributions to the literature. First, we aim to bridge the conflicting research conclusions regarding differentiated leadership. Specifically, drawing from self-verification theory (Swann, 1983), we want to make an attempt to explore the curvilinear relationship between differentiated leadership (i.e., differentiated empowering leadership) and team outcome (i.e., relationship conflict), and subsequent negative team member behavior (i.e., CWBI). By doing so, our research may provide a novel perspective for future differentiated leadership research.

Second, we extend team leadership research by studying the conditional effect of team competence variance to answer the call to study “team leadership in context” (Day et al., 2006, p. 213). Team leadership does not happen in a vacuum. As a type of team leadership, the influence of differentiated leadership can be amplified or attenuated by contextual factors (Anand et al., 2015). Hence, examining team competence variance as a boundary condition for the influence of differentiated empowering leadership on relationship conflict can provide a more comprehensive understanding of how team leadership works in a team context.

**FIGURE 1:**
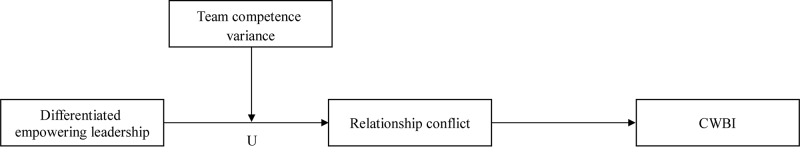
Theoretical model. CWBI is short for counterproductive work behaviors toward individuals.

Third, we contribute to a burgeoning stream of research examining the influence of empowering leadership in the team context (e.g., Li et al., 2015; Li N. et al., 2017). Specifically, we do not simply apply a dyadic or individual perspective of empowering leadership theory in the team context. Instead, we explore the influence of differentiated empowering leadership to depict the picture of the interaction process between empowering leaders and team members and among team members. We also contribute to the empowering leadership literature by answering the call to explore the negative outcomes of empowering leadership (Sharma and Kirkman, 2015). We explored whether, how, and when differentiated empowering leadership might influence relationship conflict and CWBI. Hence, this research also broadens the literature on the relationship between positive leadership, relationship conflict, and CWBI. The overall theoretical model is presented in Figure 1.

## Theory and Hypotheses

### Differentiated Empowering Leadership

The idea of differentiated leadership was first elaborated in the LMX literature (Liden et al., 2006; Erdogan and Bauer, 2010) and was later extended to the research on transformational leadership (Wu et al., 2010; Cole et al., 2011), laissez-faire leadership (Cole and Bedeian, 2007), and empowering leadership (Li et al., 2015; Li S.L. et al., 2017). Among the types of differentiated leadership, we focus on differentiated empowering leadership for two reasons. First, with the rapid transition of society from a production economy to an Internet economy, leaders can no longer only rely on their skills, knowledge, and experience to respond to the rapidly changing business environment (Lovelace et al., 2007). Meanwhile, with a new generation (people born after 1980) flooding the job market, employees are increasingly eager for autonomy and participation in decision-making, leading to the widespread adoption of empowering leadership in numerous companies, such as Google, Microsoft, and LinkedIn. In the past decade, therefore, empowering leadership has drawn increasing interest from scholars (Sharma and Kirkman, 2015).

Second, Forrester (2000) claimed that “one-size-fits-all empowerment” might result in detrimental outcomes because not all employees are ready or want to be empowered. Therefore, the question arises as to what happens when a team leader empowers team members differently based on personal characteristics such as competence. We followed Li et al. (2015), Li S.L. et al. (2017) and defined differentiated empowering leadership as the degree to which team leaders provide different amounts of authority and autonomy to team members.

### The Curvilinear Relation Between Differentiated Empowering Leadership and Relationship Conflict

#### Linking Self-Verification Theory With Differentiated Empowering Leadership

Self-verification theory assumes that individuals want to maintain self-congruence (Swann, 1983). A congruent self-identity affords individuals a sense of certainty and composure (Swann et al., 2002). Hence, individuals want others to confirm and thus stabilize their self-identity (Swann, 1983). In a nutshell, people expect others to see them as they see themselves. Whether an individual’s self-views are verified will influence their behaviors and interaction patterns (Swann, 1983). Specifically, individuals sense that being understood by others will ease social interactions and that being misunderstood will create social turbulence (Swann et al., 2000).

In a team context, a critical way for a team member to ascertain whether others’ appraisals confirm their self-view is through signs and symbols from the team (Swann et al., 2004). Specifically, a member is more concerned about the team leader’s evaluation than other team members because the leader is the critical performance appraiser and is in charge of the team (Scott and Einstein, 2001; Zaccaro et al., 2009). Accordingly, the leader is a key signaler for team members to determine whether their self-conceptions are confirmed.

Self-views of competence are vital for employees because the desire for competence takes on significant importance in organizational settings (Deci and Ryan, 1980; Bandura, 1986). When a team leader empowers the team members in different ways, team members will surmise that this difference reflects the leader’s evaluation of their competence based on the assumption that leaders give more authority and autonomy to trusted, competent team members (Hakimi et al., 2010). Hence, differentiated empowering leadership can be perceived as an important symbol for team members to verify their competence self-views and may further influence their social interactions (Swann, 1983). We discuss the influences of differentiated empowering leadership on team member self-verification and further on relationship conflict which is the result of social interaction and defined as interpersonal incompatibility accompanied by tension, friction, annoyance, and frustration (Pelled et al., 1999).

Based on the recommendation of Harrison and Klein (2007), we simplified the levels of differentiated empowering leadership into three general patterns: low, medium, and high. We speculate that relationship conflict within a team will be high if some team members are not self-verified and behave inappropriately when differentiated empowering leadership is low or high. However, with a medium level of differentiated empowering leadership, relationship conflict will be low because team members may feel understood and interact more appropriately (Swann et al., 2000; Thatcher and Greer, 2008).

#### From a Low to a Medium Level of Differentiated Empowering Leadership

Regarding a low level of differentiated empowering leadership, we simply speculate that there are three sub-patterns. Specifically, when a team leader shares a vast amount of authority with all team members, the extent of differentiated empowering leadership is low (Harrison and Klein, 2007). Although several team members who hold a self-view of high competence will feel self-verified because of the high empowerment, team members who are not confident in their competence may not feel understood and may regard the high empowerment as an unreasonable demand on them (Spreitzer and Doneson, 2005; Hakimi et al., 2010; Cheong et al., 2016; Wong and Giessner, 2018). Several self-verification researchers have contended that people want to be seen as they see themselves—even if that means others recognizing their flaws and limitations (Swann et al., 2000). Swann et al. (1989) argued that when people hold negative self-views, they will strive to eschew positive feedback in favor of negative, but verifying, feedback.

A typical way to elicit self-confirmatory reactions, drawn from self-verification theory, is to display identity cues that can be noticed by others (Swann et al., 2004). Because a superior in the team has legitimate authority (e.g., promotion decision and performance appraisal), team members sometimes must tolerate incongruent appraisals from their leader (Swann et al., 2009). However, team members may choose to compensate for a non-verifying appraisal from their leader by cultivating self-verification from coworkers (Swann et al., 2009). Team members who lack self-confidence may withdraw and perform poorly to make others see them as less competent (Thatcher and Greer, 2008). Similarly, Swann and Hill (1982) found that individuals who consider themselves submissive will behave more submissively when interacting with a person who views them as dominant. However, irresponsible behavior by low competence members can lead to dissatisfaction for other members because of team performance pressure, which in turn leads to friction and relationship conflict (Jehn and Mannix, 2001; De Dreu and Weingart, 2003).

When a team leader does not share any authority with team members at all, the extent of differentiated empowering leadership is also low (Harrison and Klein, 2007). In this circumstance, team members with high self-confidence may not feel understood and want to be more empowered (Houghton and Yoho, 2005). Self-verification theory identified “interpersonal prompts” as a type of interaction strategy that individuals can adopt to acquire self-confirmatory reactions (Swann, 1983). Thus, confident team members may demonstrate their authority and involvement in the decision-making process, which may incur resentment from other team members and initiate friction and conflict within the team (Janssen, 2004).

The third sub-pattern of low differentiated empowering leadership is when a leader allocates a certain amount (neither very much nor very little) of authority to team members equally (Harrison and Klein, 2007). In this case, both high- and low-confidence team members may not feel self-verified. A medium level of empowerment cannot satisfy the high authority needs of highly confident team members, while it may still be too much for team members lacking confidence (Humborstad et al., 2014). Accordingly, high-confidence team members will show their authority radically, and low-confidence team members will flinch and perform poorly, both of which can lead to disgust from other team members and induce relationship conflict (Litrico and Choi, 2013). Because stable and verified self-views can make people more predictable by stabilizing their behavior (Goffman, 1959), Thus, the predictability of team members’ behavior may be weakened and the probability of tension and annoyance within a team increased. In sum, a low level of differentiated empowering leadership can induce high relationship conflict within a team.

When the degree of differentiated empowering leadership increases to a medium level, team leaders neither empower team members equally nor greatly widen the authority gap between them (Harrison and Klein, 2007). They provide different but appropriate amounts of authority and autonomy to individual team members. In such a case, confident team members get enough empowerment to fit their self-views of competence, and correspondingly, low-confidence team members get empowerment that does not make them feel overloaded and stressed (Litrico and Choi, 2013; Humborstad et al., 2014). All of the team members may perceive that they are treated in a self-congruent manner, which can “promote the perceptions of coherence and calm the waters of social interaction” (Swann et al., 2000, p. 239). Indeed, according to self-verification theory, “the tendency for group members to bring others see them as they see themselves should make people feel more connected to their group” (Swann et al., 2000, p. 240). Hence, when differentiated empowering leadership increases to an intermediate level, team members can behave more appropriately in interactions, causing less friction and conflict.

To summarize, within the range from relatively low to medium levels of differentiated empowering leadership, team members will increasingly feel that they are treated congruently with their self-concept (i.e., self-competence in this study). Accordingly, within this range, as differentiated empowering leadership increases, team members are more likely to interact appropriately and less likely to experience relationship conflict.

#### From a Medium to a High Level of Differentiated Empowering Leadership

Once the degree of differentiated empowering leadership increases beyond a certain point, we assume that team members will increasingly feel misunderstood and social interaction will worsen again. Specifically, when the degree of differentiated empowering leadership increases to a relatively high level, team leaders provide dramatically different amounts of authority to individual team members and considerably widen the power gap between them (Harrison and Klein, 2007). In this case, even highly confident team members may feel overvalued and overstressed because of unreasonable expectations and authorizations accompanied by overwhelming responsibilities (Humborstad et al., 2014). Thus, they may withdraw slightly to make others see them as competent but not as supermen.

Conversely, low-confidence team members may feel that they are seriously underestimated and even humiliated (Humborstad et al., 2014). Thus, they may engage in aggressive or even *ultra vires* behaviors to show others that they deserve more empowerment (Litrico and Choi, 2013). The behavior of team members who are not self-verified may become unpredictable and “trigger the interpersonal equivalent of a devastating tidal wave” (Swann et al., 2000, p. 239). Therefore, relationship conflict will become severe within a team under a high level of differentiated empowering leadership.

Current self-verification research provides partial evidence for the relationship between differentiated leadership and relationship conflict within a team. For example, Swann et al. (2000) found that self-verification can enhance feelings of connectedness among group members and reduce relationship conflict. Moreover, Polzer et al. (2002, p. 298) examined the moderating role of interpersonal congruence drawn from self-verification theory and defined it as “the degree to which group members see others in the group as others see themselves.” Their results showed that a high level of interpersonal congruence could attenuate the positive influence of group diversity on relationship conflict. Taken together, we propose:

Hypothesis 1: Differentiated empowering leadership has a curvilinear (U-shaped) effect on relationship conflict within a team, such that the differentiated empowering leadership negatively affects relationship conflict up to a specific point (a medium level of differentiated empowering leadership); beyond that point, the relationship between differentiated empowering leadership and relationship conflict becomes positive.

### The Moderating Role of Team Competence Variety

Several variables can moderate the magnitude of self-verification effects. Whether a specific self-identity is salient or active can play such a moderating role (Lord et al., 1999; Swann et al., 2004). Situated identity, which is akin to “working self-concept,” is the currently active and salient portion of one’s identity (Lord and Brown, 2004; Swann et al., 2009). According to self-verification theory, a specific circumscribed context or situation can activate a certain situated identity (Swann et al., 2009). Specifically, in work groups or a team context, the salient identity can be triggered by examining similarities and differences (e.g., numerical distinctiveness) among members of a group or a team (Festinger, 1954; Robinson and O’Leary-Kelly, 1998).

We assume that the variance in competence within a team can moderate the curvilinear relationship between differentiated empowering leadership and relationship conflict by triggering the salience of team members’ competence identity in the self-verification process. The composition of team member characteristics is a key contextual factor that can influence the effects of leadership (Oc, 2018). Team competence variance refers to the extent of distinctiveness in team members’ competence levels. Specifically, a high degree of team competence variance means that team members have significantly different competence levels. Conversely, a low degree of team competence variance means that team members have similar competence levels (Harrison and Klein, 2007).

When competence variance within a team is high, the competence self-identity of team members can be salient and active (Deaux, 1996; Lord et al., 1999). In this circumstance, the competence self-identity becomes the situated identity (Lord and Brown, 2004). Among self-identities, self-competence can be a significantly important self-identity that team members want verified. They will be more concerned about signals and cues regarding competence appraisal, which can be acquired through a leader’s differentiated empowerment (Lord et al., 1999). In a team with a high or low level of differentiated empowering leadership, team members whose competence self-identity cannot be verified may interact more inappropriately and cause more relationship conflict when team competence variance is high rather than low (Lord et al., 1999; Thatcher and Greer, 2008). Thus, high team competence variance will amplify the negative relationship between differentiated empowering leadership and relationship conflict when the degree of differentiated empowering leadership ranges from relatively low to intermediate (i.e., the left tail of the U-shaped relation). Meanwhile, high team competence variance will amplify the positive relationship between differentiated empowering leadership and relationship conflict when the degree of differentiated empowering leadership ranges from intermediate to relatively high (i.e., the right tail of the U-shaped relation).

However, when team competence variance is low, team members’ competence self-identity may not be activated and is not salient (Lord et al., 1999; Tsui and Gutek, 1999; Thatcher and Zhu, 2006). Hence, competence self-identity is not a situated identity that team members are eager to have verified when team competence variance is low (Higgins, 1989; Lord and Brown, 2004). Team members will pay less attention to competence information drawn from a leader’s differentiated empowerment levels (Lord et al., 1999). Low team competence variance thus attenuates the negative relation between differentiated empowering leadership and relationship conflict when the degree of differentiated empowering leadership ranges from relatively low to intermediate (i.e., the left tail of the U-shaped relation). Low team competence variance lessens the positive relationship between differentiated empowering leadership and relationship conflict when the degree of differentiated empowering leadership in a team ranges from intermediate to relatively high (i.e., the right tail of the U-shaped relation). In sum, we propose:

Hypothesis 2: Team competence variance moderates the curvilinear relation between differentiated empowering leadership and relationship conflict. Specifically, the curvilinear relation between differentiated empowering leadership and relationship conflict is more significant in teams with a high level of competence variance than in teams with a low level of competence variance.

### The Curvilinear Mediated Moderation Effect on CWBI

Thus far, we have reasoned that high or low levels of differentiated empowering leadership and high levels of team competence variance jointly increase relationship conflict. We now develop the idea that high levels of relationship conflict may lead to CWBI (“individuals” refers to coworkers within a team in this study). CWB is defined as voluntary behaviors that violate organizational norms and harm the legitimate interests of the organization (i.e., CWBO), its members (CWBI), or both (Robinson and Bennett, 1995; Dalal et al., 2009). Relationship conflict exists within a team when there are interpersonal problems and incompatibilities among team members (Jehn, 1995), which is more likely to induce harm toward coworkers than the organization (Bruk-Lee and Spector, 2006). Thus, we only investigate the downstream effects of relationship conflict on CWBI.

According to the job stressor model of CWB (Spector and Fox, 2002), when employees confront stressors in the workplace, they may experience negative emotions such as anger and frustration and subsequently engage in CWB. In a team context, when there is a high level of relationship conflict, team members can feel tension, animosity, and annoyance (Jehn, 1995). To release such feelings, team members may engage in tit for tat and other negative behaviors (Spector and Fox, 2002). For example, when team members feel tense, they may try to avoid interacting. When animosity is high among a team, they may speak poorly about each other or criticize opinions or suggestions without considering whether they might be helpful. All of these negative behaviors can be categorized as CWBI. Previous research found a mean correlation between interpersonal conflict and interpersonal aggression (correlation = 0.38, Hershcovis et al., 2007). In addition, Bruk-Lee and Spector (2006) found that interpersonal conflict with coworkers might induce CWB toward other employees. Thus, we hypothesize as follows:

Hypothesis 3a: Relationship conflict within a team is positively related to its team members’ CWBI.

The relationship between positive leadership and CWB has been understudied (Kessler et al., 2013). We assume that in a team with high competence variance, differentiated empowering leadership has a curvilinear indirect effect on team members’ CWBI through relationship conflict. Team members want their self-views of competence to be verified in a team with high competence variance (Thatcher and Zhu, 2006). Leaders play an important role in the psychological process of team members’ self-verification (Lord et al., 1999). As discussed above, when differentiated empowering leadership is relatively high or low, some team members will believe that their self-views of competence have not been verified and experience negative emotions such as anger. Based on the job stressor model of CWB (Spector and Fox, 2002), team members may engage in deviant behaviors to relieve their negative emotions. Litrico and Choi (2013) found that team members whose efficacy beliefs were not recognized by others were more likely to engage in process hindrance, a kind of counterproductive engagement with others.

As discussed above, a high level of relationship conflict exists within a team when differentiated empowering leadership is relatively high or low. A high level competence variance within a team will also strengthen the curvilinear association between differentiated empowering leadership and relationship conflict. We further argue that relationship conflict might have a downstream effect on team members’ interpersonal CWB. Integrating our reasoning in the current section with our earlier theorizing, we thus propose the following hypothesis:

Hypothesis 3b: The curvilinear interactive effects of differentiated empowering leadership and team competence variance indirectly affect team members’ CWBI through relationship conflict within a team.

## Materials and Methods

### Participants and Procedure

We collected data from full-time employees of a large construction corporation. The corporation’s headquarters are in southeastern China, while its construction projects are located throughout the country. Our survey team obtained permission for the survey from a top manager. Because we wanted to explore the effects of differentiated empowering leadership in the team context, we restricted our data collection to employees who worked in the construction technician teams. They are numerous and crucial to construction quality.

We conducted a time-lagged, paper-based survey using traditional mailing because the construction technician teams were in various cities. At time 1, we sent a package containing sealed member questionnaires to each construction technician team. In the questionnaires, we asked team members to report their competence level, their leader’s empowering behaviors, and some demographic information. Before the survey began, we compiled a team-ID (e.g., No. 1) for each team and asked each team leader to compile a member-ID (e.g., No. 1-1) for each member. Only the team leader and we knew who corresponded to each member-ID, and the member-ID was used for matching and confidentiality purpose only. To ensure confidentiality, we asked team members to independently mail their completed questionnaires to us. After 2 weeks, we sent another package (to those who responded at time 1) to collect data on relationship conflict. After another 2 weeks, we sent a sealed questionnaire to team leaders whose team members had participated in the first and second rounds of the survey. In this questionnaire, we asked each team leader to evaluate his team members’ CWBI and report some background information (e.g., team size) about his team. Each team member and leader was compensated with 15 Chinese *yuan* (approximately US$2.15) for their participation in the survey.

All team members (*N* = 336) and leaders (*N* = 89) were invited to participate in the survey. At time 1, we received 318 completed questionnaires from team members nested in 86 construction technician teams. Eight team members nested in four teams were excluded because the response rate of their teams was less than 60% (Timmerman, 2005). At time 2, we received 305 responses from team members nested in 81 teams. Two team members from one team were excluded because the response rate of their team was less than 60%. At time 3, we received 78 completed questionnaires from team leaders. Two teams (with six team members) were excluded because their leaders did not respond. Hence, our final sample was 297 team members nested in 78 teams. The final response rate was 88.39%. The mean age of the 297 team members was 31.40 (*SD* = 7.52), and none were female. The mean job tenure of the participants was 5.87 years (*SD* = 5.76), and most of them had finished a college education (67.30%). The mean size of the 78 teams was 5.36 members (*SD* = 0.76). For more information of the team size, please see the Table A1 in the Appendix.

### Measures

All scale items underwent a back-translation process (c.f. Brislin, 1980) to ensure the internal validity of our translated scales.

#### Differentiated Empowering Leadership

According to the recommendation of Harrison and Klein (2007) and based on the measure of differentiated leadership by Wu et al. (2010), we used the within-team coefficient of variance (CV) to measure differentiated empowering leadership. Specifically, differentiated empowering leadership was calculated by dividing the within-team standard deviation of empowering leadership by the mean empowering leadership score of all team members. Empowering leadership was measured with the 12 items developed by Ahearne et al. (2005). A sample item is “My team leader makes many decision together with me” (1 = strongly disagree and 7 = strongly agree; α = 0.96).

#### Relationship Conflict

Team members reported their perception of relationship conflict within their teams using Tjosvold et al.’s (2006) four-item scale. Based on Chan’s (1998) typology of composition models, we used a referent shift consensus model to measure relationship conflict within each team. A sample item is “How much friction is there among members of your team?” (1 = not at all and 7 = very much; α = 0.96). Team-level relationship conflict was calculated by averaging all team members’ perceptions. Inter-rater agreement and reliability and intraclass correlation (AD_M_ = 1.18, mean *Rwg* = 0.88, *ICC*[1] = 0.53, *ICC*[2] = 0.81) justified the aggregation of relationship conflict.

#### Team Competence Variance

We used the within-team CV to measure team competence variance. Specifically, competence variance within a team was calculated by dividing the within-team standard deviation of competence by the mean competence score of all team members. Competence was measured with the three-item competence subscale of the psychological empowerment scale developed by Spreitzer (1995). A sample item is “I have mastered the skills necessary for my job” (1 = strongly disagree and 7 = strongly agree; α = 0.92).

#### Counterproductive Work Behaviors Toward Individuals

Team leaders evaluated their members’ CWBI using the six-item scale developed by Dalal et al. (2009). A sample item is “This team member behaved in an unpleasant manner toward other coworkers” (1 = strongly disagree and 7 = strongly agree; α = 0.93).

#### Controls

In line with previous research on differentiated leadership (e.g., Erdogan and Bauer, 2010), mean empowering leadership was controlled for within each team to ensure that any observed effects of differentiated empowering leadership on outcomes were not due to the influence of team-level mean empowering leadership. Based on Chan’s (1998) typology of composition models, we used an additive model to directly average the perceived empowering leadership scores of all team members. The empowering leadership measure was introduced above.

## Results

### Preliminary Analysis

Means, standard deviations, reliabilities, and correlations are presented in Table 1. A multilevel confirmatory factor analysis (MCFA) was conducted to confirm the hypothesized four-factor structure (for latent variables only) of empowering leadership, relationship conflict, competence, and CWBI, while also accounting for the between-organization variances in these latent variables. Before conducting MCFA, and in line with prior studies (e.g., Zhang and Bartol, 2010), empowering leadership was packed into four parcels representing four distinct sub-dimensions: enhancing the meaningfulness of work, fostering participation in decision-making, expressing confidence in high performance, and providing autonomy from bureaucratic constraints.

**TABLE 1:** Descriptive statistics and correlations among variables.

**Variables**	***Mean***	***SDt***	***SDi***	**Correlations**				
				
				**1**	**2**	**3**	**4**	**5**
(1) Mean empowering leadership	4.75	1.08	—	(0.96)				
(2) Differentiated empowering leadership	0.15	0.14	—	–0.52^∗∗^	—			
(3) Relationship conflict	2.47	1.11	—	–0.30^∗∗^	0.11	(0.96)		
(4) Team competence variance	0.12	0.13	—	–0.36^∗∗^	0.70^∗∗^	0.21^∗∗^	—	
(5) CWBI	2.00	0.72	0.99	–0.19^∗∗^	0.27^∗∗^	0.33^∗∗^	0.31^∗∗^	(0.93)

**SDt* and *SDi* are standard deviations computed in the team level and individual level separately; Coefficient alpha estimates of reliability are in parentheses on the diagonal; CWBI is short for counterproductive work behavior toward individuals; ^∗∗^*p* ≤ 0.01.*

The hypothesized four-factor model demonstrated a good fit to the data: χ^2^ = 308.73, *df* = 113, *p* < 0.001, *CFI* = 0.99, *TLI* = 0.99, *RMSEA* = 0.08; *SRMR* (within) = 0.03, *SRMR* (between) = 0.00. This four-factor model fit the data better than other models. For example, it fit the data better than a three-factor model grouping relationship conflict and CWBI [χ^2^ = 8188.08, *df* = 116, *p* < 0.001, *CFI* = 0.49, *TLI* = 0.40, *RMSEA* = 0.48; *SRMR* (within) = 0.20, *SRMR* (between) = 0.00; Δχ^2^ = 377.54, Δ*df* = 3, *p* < 0.001], or another three-factor model grouping empowering leadership and competence [χ^2^ = 5587.00, *df* = 116, *p* < 0.001, *CFI* = 0.65, *TLI* = 0.59, *RMSEA* = 0.40; *SRMR* (within) = 0.14, *SRMR* (between) = 0.00; Δχ^2^ = 299.15, Δ*df* = 3, *p* < 0.001]. Overall, the results of the MCFA support the discriminant validity among our focal constructs.

### Hypotheses Testing

Given the nested structure in our model, we used multilevel path-analytical modeling to test our hypotheses. Model estimation was conducted using the Mplus 7.0 software (Muthén and Muthén, 2010). To help interpret the findings, we standardized our predictor and control variables to obtain estimates of the hypothesized relationships (Friedrich, 1982).

We conducted path-analytical models to test our hypotheses (Hypothesis 1, 2, and 3a). Hypothesis 1 proposed that differentiated empowering leadership has a U-shaped effect on relationship conflict within a team. We regressed relationship conflict on mean empowering leadership, differentiated empowering leadership, and its squared term. The results, shown in Table 2, revealed that the curvilinear relation was statistically not significant (*B* = −0.10, *SE* = 0.07, *n.s.*). Thus, Hypothesis 1 was not supported.

**TABLE 2:** Multilevel path analysis results for testing hypotheses 1 and 2.

**Variables**	**Relationship conflict**			
	
	**Model 1 (H1)**		**Model 2 (H2)**	
		
	**Estimate**	***SE***	**Estimate**	***SE***
Intercept	2.37^∗∗∗^	0.14	2.48^∗∗∗^	0.16
Control variable				
Mean empowering leadership	–0.33^∗∗^	0.12	–0.38^∗∗∗^	0.11
Predictors				
Differentiated empowering leadership	–0.23	0.18	–0.43	0.22
Differentiated empowering leadership squared	0.10	0.07	0.15	0.13
Moderating effect				
Team competence variance			0.41	0.25
Differentiated empowering leadership × Team competence variance			−0.54^∗^	0.26
Differentiated empowering leadership squared × Team competence variance			0.11^∗^	0.05
Residual variance	1.08^∗∗∗^	0.20	0.99^∗∗∗^	0.16

**N* = 297 at the individual level, *N* = 78 at the team level; ^∗^*p* ≤ 0.05, ^∗∗^*p* ≤ 0.01, ^∗∗∗^*p* ≤ 0.001.*

Hypothesis 2 proposed that team competence variance would moderate the curvilinear relationship between differentiated empowering leadership and relationship conflict. As shown in Model 2 of Table 2, we further introduced team competence variance, the product terms between team competence variance and differentiated empowering leadership and between team competence variance and squared differentiated empowering leadership, into the analysis. The results indicated that team competence variance significantly interacted with differentiated empowering leadership (*B* = −0.54, *SE* = 0.26, *p* < 0.05) and squared differentiated empowering leadership (*B* = 0.11, *SE* = 0.05, *p* < 0.05) to influence relationship conflict. Hence, Hypothesis 2 was supported.

**FIGURE 2:**
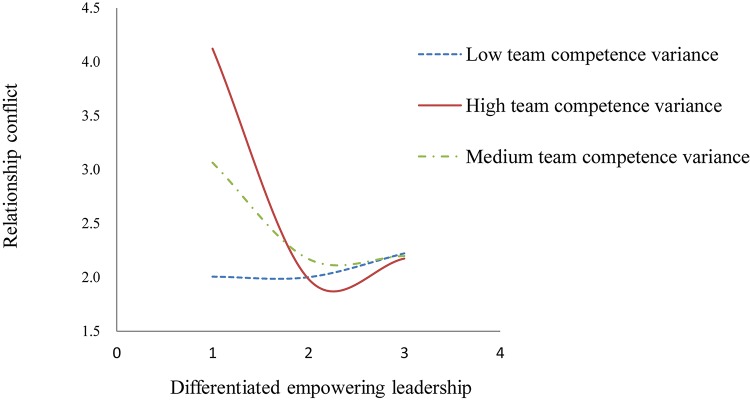
Curvilinear relationship between differentiated empowering leadership and relationship conflict as a function of team competence variance.

To further test Hypothesis 2, we followed Cohen, Cohen et al.’s (2003) procedure to plot the simple main effects in Figure 2. As can be seen in Figure 2, in a team with high competence variance, relationship conflict decreased sharply when differentiated empowering leadership increased from a low to a medium level. Moreover, when the increasing level of differentiated empowering leadership exceeded the inflection point, relationship conflict within a team gradually increased again. However, in a team with low competence variance, the curvilinear relationship between differentiated empowering leadership and relationship conflict seemed not to exist.

Hypothesis 3 involved the curvilinear mediated moderation effect on team members’ CWBI. We included relationship conflict as the mediator between the interactive curvilinear effect of differentiated empowering leadership and team competence variance and CWBI at the same time in the path analysis. As shown in Model 2 of Table 3, relationship conflict was significantly related to CWBI (*B* = 0.25, *SE* = 0.10, *p* < 0.01), and thus Hypothesis 3a was supported. To calculate the curvilinear indirect effect for Hypothesis 3b, we followed the procedures introduced by Hayes and Preacher (2010) and modified and used by Lin et al. (2017). Specifically, we state the procedures for testing curvilinear indirect effects in detail as follows.

**TABLE 3:** Multilevel path analysis results for testing hypotheses 3a.

**Variables**	**CWBI**			
	
	**Model 1**		**Model 2 (H3a)**	
		
	**Estimate**	***SE***	**Estimate**	***SE***
Intercept	2.06^∗∗∗^	0.12	1.43^∗∗∗^	0.24
Control variable				
Mean empowering leadership	–0.07	0.09	0.02	0.09
Predictors				
Differentiated empowering leadership	0.02	0.15	0.13	0.13
Differentiated empowering leadership squared	–0.01	0.09	–0.05	0.09
Moderating effect				
Team competence variance	0.22	0.19	0.11	0.15
Differentiated empowering leadership × Team competence variance	–0.29	0.20	–0.15	0.14
Differentiated empowering leadership squared × Team competence variance	0.09^∗^	0.04	0.06	0.03
Indirect effect				
Relationship conflict			0.25^∗∗^	0.10
Residual variance	0.37^∗∗∗^	0.11	0.31^∗∗∗^	0.08

**N* = 297 at the individual level, *N* = 78 at the team level; CWBI is short for counterproductive work behavior toward individuals; ^∗^*p* ≤ 0.05, ^∗∗^*p* ≤ 0.01, ^∗∗∗^*p* ≤ 0.001.*

Before introducing curvilinear mediated moderation, we first define a curvilinear mediation effect. Hayes and Preacher (2010) explained that a curvilinear mediation effect is a particular case of an indirect effect in which an independent variable is non-linearly associated with a mediator, and in turn, linearly related to a dependent variable. They used θ to denote the rate at which a change in the independent variable changes the dependent variable indirectly through changes in the mediator. To calculate θ, we find the product of the first partial derivation of the function of the mediator with respect to the independent variable and the first partial derivation of the function of the dependent variable with respect to the mediator. For this study, the formula can be described as:

(1) $$\theta =\frac{\partial \left(r\,e\,l\,a\,t\,i\,o\,n\,s\,h\,i\,p\,c\,o\,n\,f\,l\,i\,c\,t\right)}{\partial \left(d\,i\,f\,f\,e\,r\,e\,n\,t\,i\,a\,t\,e\,d\,e\,m\,p\,o\,w\,e\,r\,i\,n\,g\,l\,e\,a\,d\,e\,r\,s\,h\,i\,p\right)}\,\;\,\frac{\partial \left(C\,W\,B\,I\right)}{\partial \left(r\,e\,l\,a\,t\,i\,o\,n\,s\,h\,i\,p\,c\,o\,n\,f\,l\,i\,c\,t\right)}$$

We can derive the partial derivative of relationship conflict with respect to differentiated empowering leadership from Equation (2), and likewise, derive the partial derivative of CWBI with respect to relationship conflict from Equation (3) as follows:

(2) Relationship conflict    = β0+ β1 (mean empowering leadership)          +β2 (differentiated empowering leadership)          +β3 (differentiated empowering leadership squared)          +β4 (team competence variance)          +β5 (differentiated empowering leadership   × team competence variance)          +β6 (differentiated empowering leadership squared   × team competence variance) + σ

(3) $$\begin{array}{l} CWBI\\ \;=\;{\text{β}}_{7}+\;{\text{β}}_{8}\;(mean\;empowering\;leadership)\\ \;+{\text{β}}_{9}\;(differentiated\;empowering\;leadership)\\ \;+{\text{β}}_{10}\;(differentiated\;empowering\;leadership\;squared)\\ \;+{\text{β}}_{11}\;(team\;competence\;\mathrm{var}iance)\\ \;+{\text{β}}_{12}\;(differentiated\;empowering\;leadership\\ \;\times \text{ team competence variance})\\ \;+{\text{β}}_{13}\;(differentiated\;empowering\;leadership\;squared\\ \begin{array}{l} \;\times \text{ team competence variance})\\ \;+{\text{β}}_{14}\;(relationship\;conflict)+\sigma \end{array} \end{array}$$

According to Equations (1), (2), and (3), the instantaneous indirect effect of differentiated empowering leadership–team competence variance on CWBI through relationship conflict is

(4) $$\begin{array}{l} {\text{θ = [β}}_{2}+\;2{\text{β}}_{3}\;(differentiated\;empowering\;leadership)\\ \;+{\text{β}}_{5}\;(\text{team competence variance})\\ \;+2{\text{β}}_{6}\;(differentiated\;empowering\;leadership\\ \;\times \text{ team competence variance})]\;\times \;{\text{β}}_{14}\; \end{array}$$

As shown in Equation (4), θ is a linear function of differentiated empowering leadership, team competence variance, and the product term (differentiated empowering leadership × team competence variance). Similar to the rationale of curvilinear mediated moderation used by Lin et al. (2017), if the difference in θ at low versus high levels of differentiated empowering leadership and team competence variance is significantly different from zero, then Hypothesis 3b of curvilinear mediated moderation effect will be supported. Specifically, as bootstrapping is not available for multilevel modeling in Mplus software (Muthén and Muthén, 2010), we followed the Monte Carlo method and used R software to calculate the bias-corrected bootstrap 95% confidence intervals for the curvilinear indirect relationships between differentiated empowering leadership and CWBI via relationship conflict at high and low levels of team competence variance.

With 20,000 resamples in R program, the bootstrapping results showed that the difference in θ for low levels of differentiated empowering leadership when team competence variance was high versus low was −0.386 (95% CI = [−0.872, −0.051]), which did not contain zero. However, the difference in θ for high levels of differentiated empowering leadership when team competence variance was at high versus low levels was −0.160 (95% CI = [−0.523, 0.113]), which contained zero. In addition, the confidence interval for the two difference scores was −0.226 (95% CI = [−0.567, −0.004]), which did not contain zero. These results supported our hypothesis that team competence variance moderated the curvilinear effects of differentiated empowering leadership on CWBI through relationship conflict as an intervening variable. Thus, Hypothesis 3b was supported.

## Discussion

Drawing on self-verification theory, we developed and tested a multilevel model to examine whether, how, and when differentiated empowering leadership has a curvilinear effect on relationship conflict within a team and influences team members’ CWBI. Specifically, empirical results from a multi-wave and multi-source study showed that the curvilinear relationship between differentiated empowering leadership and relationship conflict was not significant. We speculate that the major reason for this non-significant result is that self-views of competence are not activated in teams with a low level of competence variance. Although employees’ self-views of competence are important because the desire for competence is important in the work setting (Deci and Ryan, 1980; Bandura, 1986), it may not be the salient self-identity that they want verified in teams with low competence variance (Lord et al., 1999; Swann et al., 2004). Our study supported that when competence variance among team members was high, differentiated empowering leadership had a U-shaped effect on relationship conflict and had a further effect on CWBI. However, when competence variance among team members was low, the aforementioned influence of differentiated empowering leadership did not exist.

### Theoretical Contributions

Several theoretical contributions are worth highlighting. First, we bridged the conflicting research conclusions regarding differentiated leadership by exploring the curvilinear influences of differentiated empowering leadership. There are two major research streams in the literature on differentiated leadership. Specifically, drawing on a fairness perspective (e.g., relative deprivation theory), several researchers found that differentiated leadership had detrimental influences on individual attitudes and behaviors or team interaction and effectiveness (e.g., Hooper and Martin, 2008; Wu et al., 2010). However, based on role theory, several researchers have argued that differentiated leadership might help increase individual and group performance in some circumstances (e.g., high task interdependence within the group) (e.g., Liden et al., 2006).

We tried to fill the gap and advance the research on differentiated leadership from a new perspective, namely the self-verification perspective. Differentiated leadership could be a signal that triggers team members’ striving for self-verification, which is crucial for social interaction (Swann, 1983). Although we did not find a direct curvilinear relationship between differentiated empowering leadership and relationship conflict, the results demonstrated that this U-shaped relationship existed when team members’ need for competence self-verification was activated, which is consistent with self-verification theory. Apart from the role assignment and justice perspectives, we found that self-identity confirmation might be another important mechanism linking differentiated leadership and outcomes. Thus, this research contributes to the literature on differentiated leadership by broadening the underlying theory for explaining how and when differentiated leadership might influence related outcomes in a non-linear way.

Second, we contribute to the literature on differentiated leadership by considering the team context. Team context, such as composition and types, varies from team to team, and it can pose challenges for understanding the different influences of team leadership. Differentiated leadership is a typical type of team leadership, and its influences on individual attitudes and behaviors or team processes and effectiveness may be dependent on team context. Several researchers have investigated the moderating role of team context in the relationship between differentiated leadership and team effectiveness. For example, Liden et al. (2006) demonstrated that task interdependence could strengthen the positive influence of LMX differentiation on group effectiveness. However, the related empirical evidence is still scarce (Anand et al., 2015).

We answered Day et al. (2006, p. 213) call for “studying team leadership in context” by simultaneously examining the effects of leadership structures (i.e., differentiated empowering leadership) and team context (i.e., team competence variance). We found that in teams with a high level of competence variance among members, differentiated empowering leadership had a U-shaped effect on relationship conflict, which had a downstream effect on members’ CWBI. The results showed that team context might influence the effect of team leadership by activating team members’ specific self-identity. Thus, this study provides a new perspective for exploring the moderating role of team context in team leadership research, especially for differentiated leadership research.

Our third contribution is to the empowering leadership literature. Considering and testing leadership as a multilevel process of leader-follower interactions is essential to advancing leadership research (Yammarino, 2013), so is research on empowering leadership (Cheong et al., 2019). However, insufficient attention has been paid to empowering leadership in the team and cross-level contexts(for a review, see Cheong et al., 2019). We specifically examined the effects of a leader’s differentiated empowering behaviors within a team on the team interaction process (i.e., relationship conflict), which had spillover effects on team members’ behaviors (i.e., CWBI). Hence, we advanced the literature of empowering leadership in the team context by investigating its cross-level influences and mechanisms.

We also answered the call to examine the “less positive and unintended, negative outcomes of empowering leadership” (Sharma and Kirkman, 2015, p. 194). Knowledge of the dark side of empowering leadership is limited. In the limited study that we are aware of, for example, Lee et al. (2017) drew on the too-much-of-a-good-thing perspective and explored the curvilinear relationship between empowering leadership and task performance. Based on self-verification theory, we learned more about how and when empowering leadership might induce negative outcomes by considering the different modes of a leader’s empowering behaviors within a team (i.e., leaders provide different amounts of authority and autonomy to different team members, namely differentiated empowering leadership) and their influences on disordered team interaction (i.e., relationship conflict) and team members’ harmful behaviors (i.e., CWBI).

Finally, this study broadened the literature on relationship conflict and CWBI. Although the association between relationship, or interpersonal, conflict and CWB has been firmly established (Berry et al., 2012), the relationship between leadership, especially positive leadership, and relationship conflict or CWB has been less studied (Kessler et al., 2013). This study enriched the research on the influences of positive leadership on relationship conflict and CWB by investigating the effects of differentiated empowering leadership on team members’ relationship conflict, and subsequently, on their CWBI. The results showed that the distribution and composition of leadership within a team could induce relationship conflict among team members. Thus, it extended the antecedents of relationship conflict research. In addition, our study enriched the emerging stream of CWB research that distinguishes between sources of interpersonal conflict (Bruk-Lee and Spector, 2006). Previous research found that interpersonal conflict with a supervisor induces employees’ CWB toward the organization while interpersonal conflict with colleagues induces employees’ CWB toward coworkers (Frone, 2000; Bruk-Lee and Spector, 2006; Hershcovis and Barling, 2010). In a similar vein, our results showed that relationship conflict among team members results in CWB toward individuals.

### Practical Contributions

The results of the current research also offer some practical implications for how to lead in a team context, especially for how to empower team members. One-size-fits-all empowerment should be avoided because leaders use empowerment in different settings (Forrester, 2000, p. 69). We found that in teams with high competence variance, highly differentiated empowerment and non-differentiated empowerment were both detrimental to team interaction and could cause counterproductive responses. Accordingly, we suggest that team leaders pay more attention to the competence composition of their teams. In teams with low competence variance, the striving mechanism for competence self-verification is not activated, so team members may not care whether their competence self-identity can be verified. In that case, the detrimental effects of differentiated empowerment may not emerge. In teams with high competence variance, team members may care a lot about whether others affirm their competence self-view. A leader’s differentiated empowerment can be an essential signal, reflecting how the leader evaluates team members’ competence. In such a case, leaders should not empower team members equally or overly widen the authority gap among members to conform to members’ competence self-evaluation and induce less friction.

### Limitations and Future Directions

There are several limitations in the current research. First, although we conducted a multi-wave and multi-source study to examine our hypotheses, this did not allow us to establish a causal link. We encourage researchers to conduct experiments or collect longitudinal data to replicate our hypotheses in the future. Second, we collected the data in a single corporation, which created potential challenges to generalizability. Although our study design might rule out some organization-level contextual factors that could contaminate our findings, future research should collect data from diversified organizations to strengthen the external validity of this model.

Third, we only explored the non-linear effects of differentiated empowering leadership. Although we presumed that the other types of differentiated leadership might have similar effects, there can be nuanced differences in how and when they play their roles. Hence, future research investigating whether our findings can be applied to other types of differentiated leadership are encouraged. Fourth, we only examined a contextual boundary condition in our research framework. We encourage future research to investigate the moderating role of individual differences such as the distinct forms of self. For example, researchers can explore whether the relational self and collective self (see Lee et al., 2012 as an example) play different roles in the self-verification process intrigued by differentiated leadership in the team context.

Finally, we only tested the research framework in the Chinese cultural context. Based on the cultural self-representation model (Erez and Earley, 1993), the development of self-concept depends on cultural values. One such cultural factor is power distance, which is highly related to empowerment practices. People with high power distance value respect for and submit to authority (Loi et al., 2012). Compared to employees with low power distance value, employees with high power distance value may not suspect leaders’ empowerment practices. Hence, the moderated curvilinear relationship we studied may not apply in a society with a high level of power distance. With the economic reforms in China, the power distance value of Chinese society and people has declined (Liao J. et al., 2010). Thus, we urge future research to re-examine our research framework in a society with a relatively high power distance.

## Conclusion

Our research showed that in the teams with high competence variance, differentiated empowering leadership has a U-shaped effect on relationship conflict and, subsequently, on members’ CWBI. The underlying psychological mechanism is that in teams with high competence variance, team members’ competence self-views are activated but not verified when differentiated empowering leadership is relatively low or high. In sum, our study tried to bridge the inconsistent findings and advance the literature of differentiated leadership and empowering leadership by introducing the self-verification theory.

## Data Availability

The datasets generated for this study are available on request to the corresponding author.

## Ethics Statement

We have consulted Hubei University’s ethics committee approval to conduct this survey before starting the data collection. The ethics committee of the university has reviewed and approved our study. This study does not violate any legal regulations or common ethical guidelines based on our research design. We introduced our research purpose, goals, and plans to each participant and asked their permission to participate in this academic research.

## Author Contributions

SlL developed the research model, analyzed the data, and co-drafted the manuscript. SL provided constructive suggestions to the research design, collected the data, and co-drafted the manuscript. FS participated in the data analysis and edited the manuscript. ZG edited the manuscript. All authors listed have made a substantial, direct and intellectual contribution to the work, and approved it for publication.

## Conflict of Interest Statement

The authors declare that the research was conducted in the absence of any commercial or financial relationships that could be construed as a potential conflict of interest.

## Appendix

**TABLE A1:** Team size of the sample teams in this study.

**Team size**	**The number of teams of this team size**
3	2
4	7
5	30
6	39
